# Improving antimicrobial treatment in terms of antimicrobial stewardship and health costs by an OPAT service

**DOI:** 10.1007/s15010-024-02194-0

**Published:** 2024-02-29

**Authors:** Andrea R. Burch, Bruno Ledergerber, Martin Ringer, Maria Padrutt, Claudine Reiber, Fabienne Mayer, Annelies S. Zinkernagel, Nadia Eberhard, Marisa B. Kaelin, Barbara Hasse

**Affiliations:** 1 University of Basel, Basel, Switzerland; 2 Cantonal Pharmacy, University Hospital of Zurich, Spöndlistrasse 9, 8006 Zurich, Switzerland; 3 Department of Infectious Diseases and Hospital Epidemiology, University Hospital Zurich, University of Zurich, Zurich, Switzerland; 4 Department of Infectious Diseases and Hospital Epidemiology, Cantonal Hospital St. Gallen, St. Gallen, Switzerland

**Keywords:** Complicated infections, Outcome, Outpatient parenteral antimicrobial therapy, Safety, Switzerland, Efficacy, Antimicrobial stewardship, Bed days saved

## Abstract

**Purpose:**

Outpatient parenteral antimicrobial therapy (OPAT) is a standard for antimicrobial therapy internationally. With this prospective cohort study, we aimed to assess the impact of an OPAT service as part of antimicrobial stewardship (AMS) and evaluate the safety and efficiency of the program while illuminating the financial benefit for the hospital.

**Methods:**

Socio-demographic data, treatment regimen and outcomes were prospectively recorded for all patients assigned to the program of the OPAT unit of the University Hospital of Zurich between November 2018 and September 2022.

**Results:**

In total, we recorded 303 OPAT assignments of which 260 resulted in effective OPAT episodes. The 260 OPAT episodes were further optimized toward the choice of antimicrobial agent (*n* = 18) and length of therapy (*n* = 6). Moreover, OPAT resulted in alteration of patient assessment and care led by AMS strategies in 247 of 260 episodes (95%). While the bed days saved per year increased consistently with time, a total of 3934 in-hospital treatment days were saved amounting to a cost saving of 9,835,000 CHF over 47 months. Adverse events were recorded in 46 cases whilst only two of these have been the reason for readmission during OPAT treatment. Clinical cure was noted in 77% (199/260) and was negatively associated with Charlson Comorbidity Index (CCI; OR per 1 unit higher 0.85 (95% CI 0.78–0.93)).

**Conclusion:**

This study demonstrates the impact of an OPAT service in the framework of AMS as well as its benefits for the hospital whilst preserving safety and efficacy for the patient’s parenteral antimicrobial treatment.

**Supplementary Information:**

The online version contains supplementary material available at 10.1007/s15010-024-02194-0.

## Introduction

Antimicrobial therapies may be extensive and complex concerning the way of administration, length of therapy and antimicrobial resistance situation. Since the percentage of elderly, frail and comorbid patients in health care is increasing, long-term hospitalizations due to parenteral administration of antimicrobial agents are numerous (Inpatient Parenteral Antimicrobial Therapy, IPAT). However, IPAT is associated with extensive costs and an increased patient risk of acquiring nosocomial infections and experiencing adverse events [1]. Moreover, the necessity of prolonged parenteral antimicrobial therapy often exhausts the rehabilitation potential of affected patients. In contrast, Outpatient Parenteral Antimicrobial Therapy (OPAT) programs allow for the administration of intravenous antimicrobial therapy to patients in an outpatient setting thus facilitating home care and rehabilitation. OPAT can be used for various infections in situations where the patients need parenteral treatment but are otherwise stable and in no need of inpatient monitoring. These patients may be discharged early to an OPAT program or may avoid hospital admission altogether. OPAT programs are standard of care and have proven to be a safe and cost-effective alternative to IPAT [2–5].

Owing to their convenient once-daily administration, ceftriaxone and ertapenem are important pillars of many OPAT programs. Since both antimicrobial therapies have a broad spectrum, there is a need for antimicrobial stewardship (AMS) and governance to combat antimicrobial resistance [6]. Although previous studies have shown good clinical outcomes for patients enrolled in OPAT programs [4, 7, 8], these results may not be generalizable to all healthcare systems. Moreover, the number of patients receiving OPAT treatment has in-creased significantly in recent years, highlighting the importance of quality measurement of antimicrobial use and of prospective monitoring. We aimed to assess the impact of an OPAT service as part of antimicrobial stewardship (AMS) and evaluate the safety and efficiency of the program for the patient while illuminating the financial benefit for the hospital (bed days saved).

## Materials and methods

### Setting

We initiated the OPAT program at the University Hospital of Zurich (USZ), Switzerland in November 2018. Before OPAT assignment, a medical consultation by an Infectious Diseases (ID) physician was recommended although not mandatory. Both OPAT program and ID consultation service are part of the hospital’s AMS program.

The OPAT team consists of a dedicated team of nurses, ID physicians, a consulting pharmacist and an economist. The quality of OPAT care is assured by the following international quality indicators (Appendix Table S1) [9]. The program is available for in and outpatients of the hospital as well as for patients referred by general practitioners and other clinics. For antimicrobial administration, three settings are available: (1) Hospital OPAT: the patient receives antimicrobial therapy in an outpatient clinic at the hospital. Dedicated hospital nursing staff take care of the patient’s antimicrobial therapy; (2) Homecare OPAT: the patient receives antimicrobial therapy at home by homecare employees; and (3) Self-administered OPAT: the antimicrobial therapy is administered by the patients themselves after previous training by an OPAT nurse. When entering our OPAT service, close clinical follow-up is mandatory and patients are seen at least once a week either in our ID department or by their treating physician.

Antimicrobial agents are preferably administered via a central line such as a peripherally inserted central venous catheter (PICC) or a port-a-cath. The choice of vascular access is dependent on the type of infusion system and the length of therapy. Antimicrobial treatment is administered by intermittent or continuous infusion, respectively. For continuous infusion, elastomeric pumps (Easypump^®^ II, B.Braun; single-use disposable device) or bat-tery-driven infusion pumps (MiniRhythmic^®^) are used to deliver pre-determined amounts of medication to the patient in a continuous manner for antibiotics with a time-depending killing mechanism. The required pressure for administrating the drug via an elastomeric pump comes from the elastomeric layer inside the pump. The pressure is consistent until the near end of the infusion. A flow restrictor in the tubing or within the elastomeric reservoir controls the accuracy of the flow rate. The pumps containing the antimicrobial are either prepared by a commercial compounder (setting: self-administered or homecare OPAT) or filled by a nurse on the ward where the filled pump is directly connected to the patient’s vascular access (setting hospital OPAT). The battery-driven infusion pumps were only used in the case of homecare-OPAT.

### Study design and participants

ZOPAT (Zurich outpatient parenteral antimicrobial therapy cohort) is a single center, prospective, open interval cohort study of patients receiving parenteral antimicrobial therapy in the context of the USZ OPAT program. Between November 2018 and September 2022, all patients older than 18 years with a signed hospital general consent (written informed consent to subsequent use of his/her personal health data for this project/research purposes) and OPAT were included in the study. Follow-up by the OPAT team was guaranteed up to one month after the stop of the last OPAT episode. The Independent Ethics Committee of Zurich approved the quality assurance study (BASEC 2020–00866).

### Data collection

We designed a relational database in Microsoft Access^®^ to prospectively collect information on the indication of antimicrobial treatment using International disease classification codes (ICD-10), Antimicrobial agents used and treatment duration as suggested by the referrer. We also collected changes in the nature and/or duration of antimicrobial agent use at the instigation of the OPAT team and the reasons for not including the patient in the OPAT program if applicable. We performed regular checks for the completeness and consistency of the data.

Once the decision for OPAT was positive, socio-demographic data namely gender, age, height and weight, comorbidities, Charlson Comorbidity Index (CCI) [10], pathogen, antimicrobial agents, mode of administration, duration of therapy and type of vascular access were prospectively recorded for all patients. We assessed outcomes at the end and 30 days after OPAT completion (adverse drug events (ADE), line-related events, readmission and clinical cure). We defined clinical cure as a composite of (1) completion of the antimicrobial treatment course in the scheduled time (2), no restart of antimicrobial treatment within 30 days and (3) no relapse of infection with the primary pathogen within 30 days.

We calculated bed days saved based on the duration of the OPAT episode. This equals the number of days, during which the patient would have been staying in the hospital without the OPAT program.

### Statistics

All analyzes were descriptive. We presented continuous variables as median and interquartile ranges. We registered categorical variables as counts and percentages. Categorical variables were compared with the chi-square test or Fisher’s exact test, and continuous variables were compared with the Wilcoxon rank-sum test. We evaluated differences between groups with logistic regressions for cure-endpoint with standard errors adjusted for clustering on patient numbers because of multiple episodes of some patients. We calculated odds ratios (OR) and confidence intervals (CI) and *p*-values of < 0.05 were interpreted as being statistically significant. We used Stata/SE 17.0 (StataCorp, College Station, TX, USA) for statistical analyzes.

## Results

**Fig. 1:**
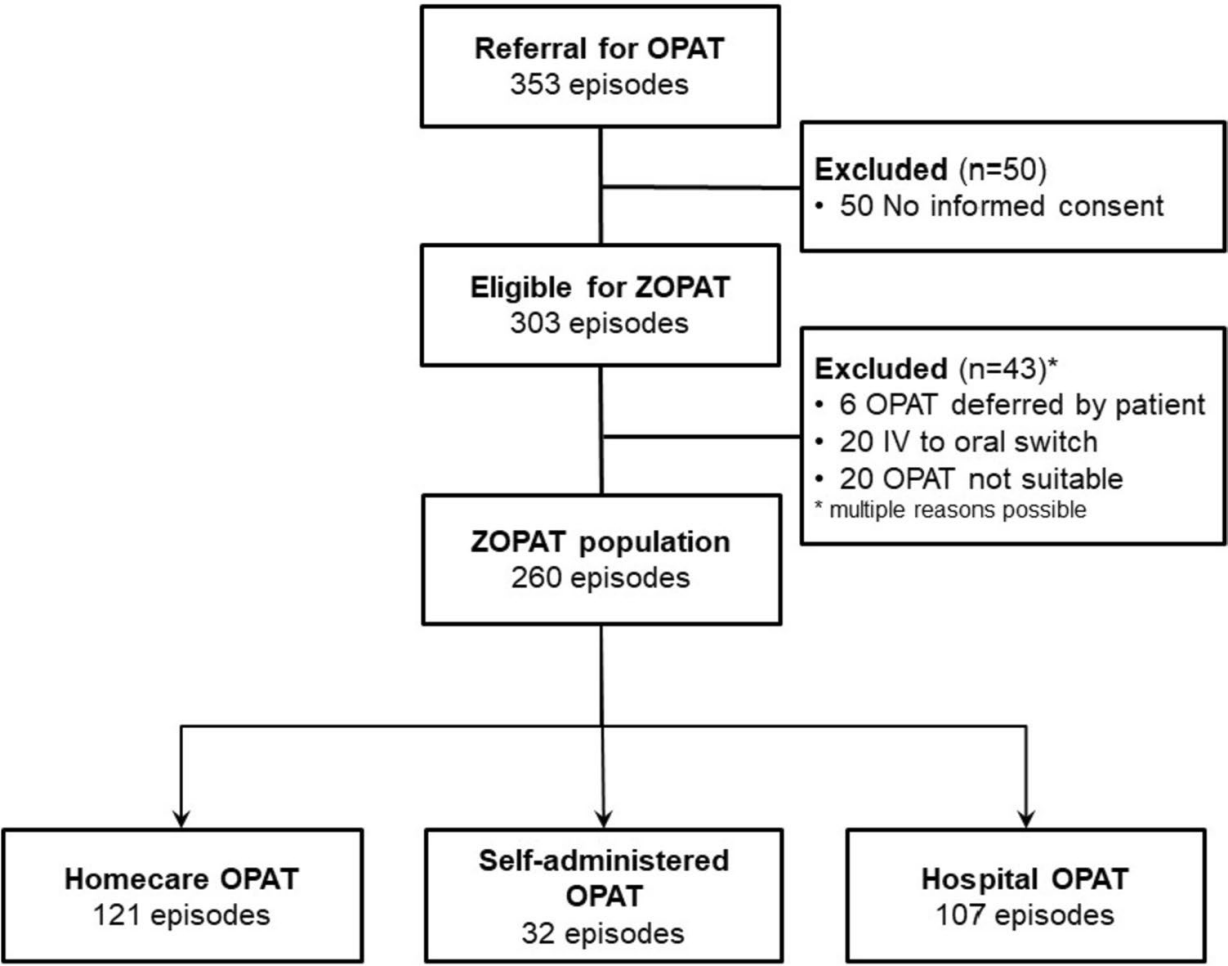
Flow chart describing the inclusion process

Between November 2018—September 2022 OPAT program enrolled 353 patients. Notably, the program permitted multiple assignments, promptin us to frame our results in the context of “episodes” rather than individual patients. Among the 353 assignments, written informed consent was secured from patients involved in 303 episodes (Fig. 1).

### Baseline characteristics

Table 1 summarizes patient characteristics (stratified to OPAT type, see Table S2). Most patients were male (*n* = 173; 67%) and the median age was 57 years (interquartile range IQR 45–68). The majority had a single OPAT episode, but 22 patients had multiple episodes. OPAT indications included 71 individual diagnoses, whereby urogenital, endovascular and osteoarticular infections constituted the main part of infections. Accordingly, Gram-negative bacteria (Enterobacterales *n* = 103; *P. aeruginosa* *n* = 38), *Staphylococcus* a*ureus* (*n* = 35), Coagulase-negative Staphylococci (*n* = 21) and *Streptococcus* spp (*n* = 17) predominated. About one-fifth of infections were polymicrobial (21%). Another important reason for OPAT was neurosyphilis (*n* = 15). The patients were referred to the program from over 26 hospital specialties. The most common antimicrobial agents included β-Lactam antibiotics (*n* = 227), followed by lipopeptides (*n* = 22).

**Table 1:** Characteristics of the patients and their treatment

Characteristics	OPAT episodes n_total_ = 260
Female sex, *n* (%)	87 (34)
Age, median years (IQR)	57 (45–68)
BMI, median kg/m^2^ (IQR)	25 (22–28)
Charlson comorbidity index, median (IQR)	3 (1–5.5)
Indication for OPAT (stratified by ICD-10 codes), *n* (%)	
Urinary tract infections	78 (30)
Foreign body associated infections, including	39 (15)
Prosthetic joint infections (*n* = 16)	
Vascular graft infections (*n* = 12)	
Breast implant infections (*n* = 2)	
Others (*n* = 9)	
Osteoarticular infections	22 (8.5)
Central nervous system infections; including:	24 (9.2)
Neurosyphilis (*n* = 15)	
Intraabdominal infections	3 (1.2)
Hepatobiliary infections	17 (6.5)
Infective endocarditis	14 (5.4)
Respiratory tract infections	9 (3.5)
Ear, nose and throat infections	5 (1.9)
Other and unspecific infections and parasitic diseases; including:	49 (18.8)
Bloodstream infections (*n* = 31)	
Antimicrobial agents used (mode of administration) *n* (%)	
Aminoglycosides	6
Antivirals	2
Antifungals	1
β-lactams	227
Cefepim (continuous) (*n* = 29)	
Cefiderocol (intermittent) (*n* = 1)	
Ceftazidim (intermittent) (*n* = 2)	
Ceftazidim/Avibactam (intermittent or continuous) (*n* = 3)	
Ceftriaxon (intermittent) (*n* = 48)	
Ertapenem (intermittent) (*n* = 57)	
Flucloxacillin (continuous) (*n* = 26)	
Meropenem (intermittent) (*n* = 8)	
Penicillin G (continuous) (*n* = 23)	
Piperacillin/Tazobactam (continuous) (*n* = 30)	
Glycopeptides (intermittent)	2
Lipopeptides (intermittent)	22
Vascular access, *n* (%)	
PICC-line or Port	190 (73)
PVC	70 (27)

An OPAT episode may have consisted of the administration of multiple antimicrobials simultaneously. Patients were allowed to be included several times in this study, by this, the data is presented as number of episodes

Outpatient parenteral antimicrobial therapy, OPAT; Peripherally inserted central catheter, PICC; Peripheral venous catheter, PVC; body mass index, BMI; Interquartile range, IQR

Most of the antimicrobials used were administered intermittently, for which no special device was needed (*n* = 158), the other 102 OPAT episodes consisted of at least one antibiotic that was infused continuously (Table 2).

**Table 2:** Mode of administration of OPAT antibiotic and duration

OPAT device	OPAT episodes *n* (%)	OPAT duration median days (IQR)	Total days of OPAT
Intermittent infusion (no device)	158 (61)	8 (5–15)	2077
Continuous infusion (elastomeric pump)	87 (33)	13 (8–26)	1483
Continuous infusion (battery-operated pump)	15 (5.8)	21 (9–32)	374
Overall	260 (100)	10 (6–21)	3934

Outpatient parenteral antimicrobial therapy, OPAT; Interquartile range, IQR

The median OPAT duration consisting of continuous infusion was longer than the duration of OPAT consisting of intermittent infusion.

### Contributions of ID consultation service and OPAT team to patient management

A total of 43 episodes were excluded from participation in the OPAT program for various reasons (with the possibility of multiple reasons occurring simultaneously). These exclusions were attributed to: OPAT being declined as a result of a possible transition from parenteral to oral treatment form (*n* = 20), a joint decision by the OPAT team and the patient’s care team that OPAT was not appropriate for the patient (*n* = 20), or the patient opting against OPAT (*n* = 6).

**Fig. 2:**
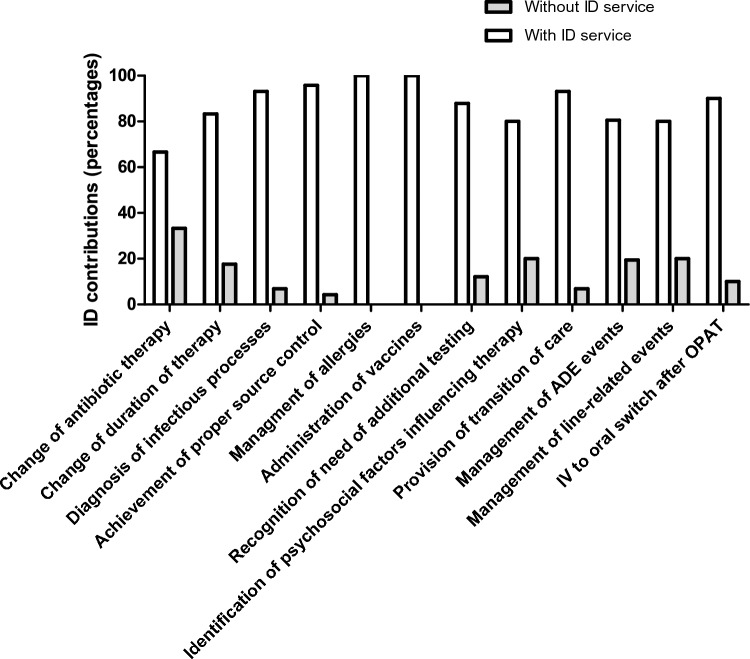
Contributions of ID consultation service and OPAT team to patient management in 260 OPAT episodes. Outpatient parenteral antimicrobial therapy, OPAT; intravenous, IV; Infectious Diseases, ID, adverse drug event, ADE

In 18 of the remaining 260 OPAT episodes, the choice of the antimicrobial treatment was adjusted and in another 6 episodes, the length of therapy was optimized (shortened *n* = 2, lengthened *n* = 4). Moreover, OPAT led to a significant alteration of patient assessment including recognition of additional testing (*n* = 141), source control (*n* = 94), allergy testing (*n* = 16), update of vaccine status (*n* = 16), recognition of a non-urgent medical problem (*n* = 33), transition of care (*n* = 247; including identification of psychosocial problems influencing treatment among 33 patients) and follow-up (intravenous (IV) to oral switch after OPAT, *n* = 50; management of line-related events or ADE in follow up visits *n* = 46). Hence, led by by ID interventions, patient care was optimized in 247 out of 260 OPAT episodes (95%) (Fig. 2).

### Safety and efficacy

Catheter-related and ADE were recorded. In 10 of 260 OPAT episodes (4%) a line-related adverse event occurred whereby ADE occurred in 36 cases (14%) (Table 3). ADE occurred in connection with β-Lactam antibiotics (ertapenem, *n* = 11; piperacillin/tazobactam, *n* = 6; penicillin, *n* = 2; flucloxacillin, *n* = 1; ceftriaxon, *n* = 4; cefepime, *n* = 3; ceftazidim, *n* = 3; cefiderocol, *n* = 1), lipopeptides (daptomycin, *n* = 3), aminoglycosides (gentamicin, *n* = 1) and antifungals (amphotericin B, *n* = 1). There was no association of ADE with length of IV- treatment (*p* = 0.81).

**Table 3:** Adverse drug-related (ADE) and line-related events. Note: Several ADEs may have occurred in one OPAT episode

Characteristics	OPAT episodes
Drug related AEs (ADEs) *n* (%)	36 (14)
Diarrhea	10
GIT complaints	9
Allergy	5
Fatigue	2
Vertigo	2
Hepatitis	2
Thrombocytopenia	2
Eosinophilia	2
Leukocytosis	1
Clostridia difficile infection	1
Drug fever	1
Taste change	1
Vaginal candidiasis	1
Hepatitis	1
Line-related events *n* (%)	10 (4)
Occlusion	5
Phlebitis	3
Dislocation	2
No drug-related AE or line-related event *n* (%)	214 (82)

Outpatient parenteral antibiotic therapy, OPAT; Gastrointestinal, GIT; adverse event, AE

Readmission of the patient during or up to 30 days after OPAT was necessary for 71 episodes (27%). Of these 71 episodes, 30% were elective readmissions and additional 25% of readmissions were due to other non-infection-related reasons. For the calculations associated with readmission, we only considered the 33 OPAT episodes that were either directly related to OPAT (*n* = 2; 1 ADE and 1 line-related complication) or exhibited clinical deterioration under therapy (*n* = 31). OPAT episodes with clinical deterioration mostly involved complex infections, where heightened comorbidity hindered achieving adequate source control, leading to a palliative therapeutic approach.

There was no association of readmissions with age (*p* = 0.38) or duration of IV treatment (*p* = 0.72). There was a trend of an association with readmission and with CCI (*p* = 0.08) and OPAT setting (*p* = 0.06), suggesting that more readmissions happened in the case of homecare-OPAT and with increasing CCI, respectively.

Clinical cure was achieved in 77% of all recorded OPAT episodes (*n* = 199). In 61 OPAT episodes, no clinical cure was achieved: (1) In 6% (*n* = 15) of OPAT episodes antimicrobial treatment was not completed as anticipated, and/or (2) re-start of antimicrobial treatment was needed in 48 cases and/or (3) a positive culture with the primary pathogen occurred in 30 OPAT episodes, respectively. The odds ratio (OR) for clinical cure was negatively associated with CCI per 1 unit higher (OR 0.85 (95% CI 0.78–0.93) and there was a trend of a negative association for age per 10 years older (OR 0.84 (95% CI 0.70–1.0) (Table 4).

**Table 4:** Cure analyzes of 260 OPAT episodes. OR calculations are based on age per 1 years older and CCI per 1 unit higher

	Total *n* = 260 (100%)	Clinical cure *n* = 199 (77%)	No clinical cure *n* = 61 (23%)	OR (95% CI)	*P*
**Age**, median years IQR	57 (45–68)	56 (44–68)	60 (48–71)	0.84 (0.70–1.0)	0.103
Charlson Comorbidity Index CCI, median IQR	3 (1–5.5)	3 (1–5)	4 (2–7)	0.85 (0.78–0.93)	0.001

Confidence Interval, CI; Odds ratio, OR; Outpatient parenteral antibiotic therapy, OPAT; interquartile range (IQR); intravenous, IV

There were no associations with female sex (OR 1.4 (95% CI 0.75–2.6), body mass index (BMI < 19 kg/m^2^: OR 2.4 (95% CI 0.27–21), BMI 18–25 kg/m^2^; reference; BMI 25–35 kg/m^2^: OR 1.2 (95% CI 0.64–2.3); BMI > 35 kg/m2: OR 0.62 (95% CI 0.18–2.1)), OPAT setting (homecare OPAT: OR 0.96 (95% CI 0.49–1.9); self-administered OPAT: OR 1.4 (95% CI 0.75–2.6); reference hospital OPAT)), venous access (peripheral catheter OR 0.76 (95% CI 0.39–1.5; reference PICC line)), and urinary tract infections (OR 0.69 (95% CI 0.36–1.3; reference all other infection types)).

### Bed days saved

**Fig. 3:**
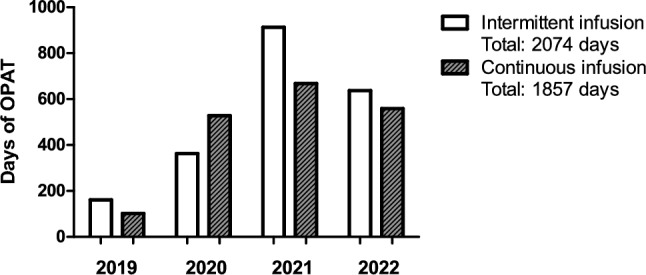
Days of OPAT stratified by intermittent versus continuous infusion therapy. Data collection censored in September 2022. Outpatient parenteral antimicrobial therapy, OPAT

The OPAT service was constantly increasing over the years delivering overall 3934 days of treatment (Fig. 3).

## Discussion

The interplay of ID consultation service, OPAT and AMS program proved to be successful toward the optimization of antibiotic use in 95% of OPAT episodes. Our study also echoes the available evidence that an efficient OPAT service enables safe homecare treatment with a substantial clinical cure rate. The OPAT program did save 3934 hospital treatment days in total, which corresponds to a median homecare treatment of 10 days.

According to estimates, approximately 30–40% of patients admitted to the hospital receive antimicrobials and thereof again 10% receive the antimicrobials intravenously. In patients where antimicrobial therapy is the main reason for hospitalization, the duration of hospitalization is the same as the duration of the IV treatment. In other patient groups, the IV antimicrobials are part of the complex medical treatment. In this case, the hospital stay is longer than the duration of the IV therapy [11]. AMS is a coherent set of actions that promote using antimicrobials responsibly [12, 13], and Infectious Diseases Society of America (IDSA) recommends measuring the appropriate use of antimicrobial agents including dosing, duration of therapy and route of administration [14]. In the first reading, AMS and OPAT thus pose a dilemma. AMS programs aim at saving antimicrobials for the future by choosing effective and safe antimicrobials with a narrow spectrum and little collateral damage, whereas OPAT programs focus on convenience, patient satisfaction, prevention of hospitalizations and early hospital discharge. What at first glance appears to be a clear contradiction may create synergies as shown in our study. AMS opportunities in our study were vast and included evaluation of the need for antimicrobial therapy, check for oral alternatives, proper advice on source control and use of narrow-spectrum antibiotics in the correct manner and dosage for the correct duration of time. Comparable to two US-based studies [15, 16], 12.4% of our patients referred for OPAT by other hospital specialists were not approved by our ID-led OPAT service. Recently, landmark studies [17–20] advocating an early switch from IV to oral therapy for complex infectious diseases have emerged, which may have influenced the decision to a priori defer OPAT treatment.

In line with the literature [16], our service provided input on the choice and duration of antimicrobial therapy in 82% of OPAT episodes. With 12.7%, our readmission rate (excluding elective hospitalizations and hospitalizations due to non-ID related events) was comparable to other reports [21], but even lower, when considering that in our study only two readmission events were related to OPAT. One reason might be, that ID follow-up was mandatory when using our service. ID specialists play a vital role in evaluating patient eligibility, selecting appropriate antimicrobials, establishing treatment plans, and monitoring patient progress and potential toxicities of Antimicrobial agents [22]. Furthermore, the ID unit acts as a resource center for healthcare providers, offering guidance on complex cases, optimizing anti-microbial use, and providing ongoing education and training. Their involvement ensures seam-less coordination between inpatient and outpatient care, facilitating the transition of patients to the OPAT program while maintaining close clinical oversight.

With 39% of all OPAT episodes, continuous infusion of narrow-spectrum antibiotics with optimal pharmacokinetic (PK)/pharmacodynamics (PD) target attainment was an important quality aspect of our ID-led OPAT service caring for patients with immunosuppression or complicated infectious diseases. As previously reported, OPAT with β-lactam antibiotics was effective [23], but antibiotic switches for ADE in our study were more frequent with continuous infusion antibiotics (23.2%) than with intermittent infusion antibiotics (13.2%). In this regard, management of complex infectious diseases should consider the risk of readmissions and ADEs in OPAT patients thus ensuring the safety and efficacy of complex OPAT patients (cOPAT) [22]. In a randomized controlled trial assessing children with cellulitis from Australia [24], home treatment with intravenous ceftriaxone was noninferior to in-hospital treatment with intravenous flucloxacillin. Like in our study, the use of ceftriaxone at home did not show an increased signal of *Clostroides difficile* colitis or antibiotic-resistant microorganisms [24, 25]. On the negative side PICC handling, drug stability, lacking license for outpatient use and costs reduce somewhat the attractiveness of continuous infusion.

The newly introduced service did save 3934 hospital bed days, which from a hospital and health care perspective is excellent and in line with the literature [26]. Assuming hypothetically that a free bed saves 2500 CHF per day, the saving costs of OPAT would have amounted to 9,835,000 CHF over 47 months. However, costs for continuous infusion are higher than for intermittent infusion therapy and a billing code for ID specialist physicians for outpatient clinical supervision is currently lacking in Switzerland. In a recent Canadian study, the introduction of a new fee-for-service payment for ID physicians did not result in a significant expansion in OPAT use [26], highlighting the fact that scaling hospital remuneration by a quality indicator that reflects the facility-specific prevalence of OPAT use should be considered. So far, OPAT staff has been mainly hired by the ID department. Since OPAT benefits the entire hospital the OPAT staff cost should be distributed across other specialties within the hospital or be funded by the hospital overhead. Therefore, policy interventions seeking to expand OPAT use should not primarily focus on ID physicians but also on other medical specialties, which would allow the inclusion of additional OPAT patients by a hospital-wide screening. The assignment of potential OPAT patients is dependent on the physician’s decision whereby we might miss many OPAT candidates.

Our study has several strengths. With the introduction of the OPAT service, a prospective data collection was started thus facilitating the assessment of clinical response to antimicrobial treatment and monitoring of adverse events related to devices and antibiotic use. Moreover, our approach with the integration of ID consultation service, OPAT and stewardship could be a role model toward the implementation of other OPAT services, emphasizing also the mandatory interprofessional approach including ID experts, nurses as well as clinical pharmacists [27, 28]. Our study has limitations. This is a monocentric Swiss study with a small sample size and therefore not generalizable to other healthcare settings. Moreover, data collection (outcomes, ADE, line-events and impact of AMS interventions) was done by the ID service. To avoid bias, a clinical pharmacist and ID physicians not involved in direct patient care adjudicated every case. However, we cannot exclude a potential documentation gap, as patients might have sought care from primary physicians or other institutions. We did not collect information on length and type of antimicrobial pretreatment. Therefore, we cannot assert whether the relatively high number of clinical failures could be influenced by the duration of pretreatment. We could not calculate associations between ADE and length of IV treatment, due to low numbers and since we did not collect the exact date of ADE.

## Conclusion

By integrating ID consultation service and OPAT in an ongoing AMS program, hospitals can effectively allocate their financial resources while ensuring high-quality care for their patients. By shifting appropriate patients from inpatient to outpatient care, OPAT not only results in a dramatic decrease in hospital stays but also brings about substantial cost reductions and an improvement in patient satisfaction underscoring the pivotal role of the proper management of antimicrobial use.

## Institutional Review Board

The study was conducted in accordance with the Declaration of Helsinki, and approved by the Independent Ethics Committee of Zurich approved the quality assurance study (BASEC 2020–00866; 23th of April 2020).

### Supplementary Information

Below is the link to the electronic supplementary material.

Supplementary file1 Table S1. Institutionalized OPAT process at the University hospital of Zurich. Table S2. Characteristics of the patients, stratified by type of OPAT (setting). DOCX 37 KB

## Data Availability

The data are not publicly available to keep confidentiality and to comply with ethical considerations. Anonymized data are available on request from the first author.
