# Supplementary material for: Improving antimicrobial treatment in terms of antimicrobial stewardship and health costs by an OPAT service
Source: Infection. 2024 Feb 29;52(4):1367–76. doi: 10.1007/s15010-024-02194-0 (PMC11289230; doi:10.1007/s15010-024-02194-0)
Supplement: Supplementary file 1 — Supplementary file1 Table S1. Institutionalized OPAT process at the University hospital of Zurich. Table S2. Characteristics of the patients, stratified by type of OPAT (setting). DOCX 37 KB [file 15010_2024_2194_MOESM1_ESM.docx]

**Supplementary Material**

**Table S1**. Institutionalized OPAT process at the University hospital of Zurich

| **Number** | **Quality indicator** |
| --- | --- |
| **0** | **Infectious Diseases consultation service** (ideally involved)   - Appropriate patient management and use of antimicrobial agents including dosing, duration of therapy and route of administration - Screening of stable patients that are hospitalized for IV antibiotic treatment |
| **1** | **Initial assessment by the OPAT Team**   - Team members: OPAT advanced practice nurses (expert in IV therapy), ID physicians (one thereof is a medically qualified lead clinician who has identified time for OPAT in his/her job plan, clinical pharmacist (counseling on antimicrobial treatment technical compounding, adapting doses and dosing intervals), economist. Ability of the patient to have OPAT: patient understands OPAT, compliance (good, moderate, bad), appropriate home environment/adequate support, IVDU, no clinical contraindications to discharge from the hospital. - Antimicrobial treatment suitable for OPAT |
| **2** | **Antimicrobial Stewardship assessment**   - Prerequisite: OPAT is part of the antimicrobial stewardship program - Decision for OPAT and appropriateness of antimicrobial substance and treatment length - If applicable: document reason why OPAT was declined: change to oral regimen possible, properties of the substance, regimen not adequate |
| **3** | **OPAT treatment and monitoring plan**   - Indication of antimicrobial treatment, type of antimicrobial treatment, dose frequency, duration of treatment, type of administration (continuous or intermittent infusion), setting (hospital OPAT, homecare OPAT, self- administered OPAT), type of vascular access device (PICC, port), follow-up plan at the hospital or elsewhere - Responsibility: ID physicians |
| **4** | **Patient information**   - Benefits, side effects, potential, complications, vascular access/sterile techniques, responsible physician until patients seen in clinic, instructions for emergencies, antimicrobial use, patient responsibilities, nature of OPAT, contact lists, use of antimicrobials (e.g. storage conditions) - Distribution of patient educational brochures in written or online form - Surveys for quality of life and patient satisfaction with OPAT |
| **5** | **Documentation**   - Clinical response to antimicrobial treatment, adverse events related to devices and antibiotic use, obtain weekly laboratory results while on treatment - Inclusion of OPAT treatment and monitoring plan in the discharge summary |
| **6** | **Drug delivery service**   - Organization by the OPAT team in accordance with homecare team (if applicable) |
| **7** | **Self-administered OPAT (if applicable)**   - Patient or carer should be trained in the administration of IV antibiotics - Patient’s or carer’s competence must be secured and documented by the OPAT nurse specialist |
| **8** | **Follow-up**   - Communication between stakeholders (general practitioner, homecare team): start and completion of antimicrobial therapy, complications - Regular, at least once weekly review of treatment plan by the OPAT specialist nurse and physician (narrow spectrum antibiotics, IV-oral switch) in conjunction/consultation with the referring specialist as necessary - In case of a clinical problem rapid low-threshold communication between the OPAT team and the patients should be ensured |
| **9** | **Outcome**   - Monitoring of program outcome of patients receiving OPAT (adverse events, mortality by infection, all-cause mortality) - Antimicrobial use - Monitoring of satisfaction and quality of life among patients receiving OPAT |
| **10** | **Vascular access**   - Institutionalized guideline for vascular access systems used including site care - Removal of intravascular access device after end of therapy if not needed for another reason |
| **11** | **Governance**   - Document that outlines the responsibilities of OPAT team members and the infection-related inclusion and exclusion criteria for OPAT |
| **12** | **Economic efficiency**   - The economist assists the OPAT team in calculating economic efficiency and communicates with hospital management |

Note: adapted after R. Stemkens *et al*. [1]

**Table S2.** Characteristics of the patients, stratified by type of OPAT (setting).

|  | **OPAT episodes** | | | |
| --- | --- | --- | --- | --- |
| **Characteristics** | **all settings**  **n=260** | **Homecare**  **n=121** | **Hospital**  **n=107** | **Self**  **N=32** |
| **Female sex**, n (%) | 87 (34) | 38 (31) | 38 (36) | 11 (34) |
| **Age**, median years (IQR) | 57 (45-68) | 60 (51-72) | 53 (40-68) | 49 (41-56) |
| **BMI**, median kg/m^2^ (IQR) | 25 (22-28) | 25 (22-28) | 25 (22-30) | 24 (22-26) |
| **Charlson comorbidity index**, median (IQR) | 3 (1-5.5) | 4 (1-7) | 3 (0-4) | 2 (0-4.5) |
| **Indication for OPAT** (stratified by ICD-10 codes), n (%) |  |  |  |  |
| Urinary tract infections | 78 (30) | 31 | 44 | 3 |
| Foreign body associated infections, including | 39 (15) | 26 | 10 | 3 |
| Prosthetic joint infections (n=16)  Vascular graft infections (n=12)  Breast implant infections (n=2)  Others (n= 9) |  |  |  |  |
| Osteoarticular infections | 22 (8.5) | 13 | 3 | 6 |
| Central nervous system infections; including: | 24 (9.2) | 9 | 8 | 7 |
| Neurosyphilis (n=15) |  |  |  |  |
| Intraabdominal infections | 3 (1.2) | 1 | 2 | 0 |
| Hepatobiliary infections | 17 (6.5) | 10 | 4 | 3 |
| Infective endocarditis | 14 (5.4) | 8 | 4 | 2 |
| Respiratory tract infections | 9 (3.5) | 4 | 3 | 2 |
| Ear, nose and throat infections | 5 (1.9) | 1 | 4 | 0 |
| Other and unspecific infections and parasitic diseases; including: | 49 (18.8) | 18 | 25 | 6 |
| Bloodstream infections (n=31) |  |  |  |  |
| **Antimicrobial agents used** (mode of administration) n (%) |  |  |  |  |
| Aminoglycosides | 6 | 2 | 4 | 0 |
| Antivirals | 2 | 0 | 1 | 1 |
| Antifungals | 1 | 0 | 1 | 0 |
| β-lactams | 227 | 110 | 88 | 29 |
| Cefepim (continuous) | 29 | 14 | 9 | 6 |
| Cefiderocol (intermittent) | 1 | 1 | 0 | 0 |
| Ceftazidim (intermittent) | 2 | 2 | 0 | 0 |
| Ceftazidim/Avibactam (intermittent or continuous) | 3 | 3 | 0 | 0 |
| Ceftriaxon (intermittent) | 48 | 23 | 24 | 1 |
| Ertapenem (intermittent) | 57 | 29 | 24 | 4 |
| Flucloxacillin (continuous) | 26 | 10 | 8 | 8 |
| Meropenem (intermittent) | 8 | 6 | 0 | 2 |
| Penicillin G (continuous) | 23 | 10 | 9 | 4 |
| Piperacillin/Tazobactam (continuous) | 30 | 12 | 14 | 4 |
| Glycopeptides (intermittent) | 2 | 2 | 0 | 0 |
| Lipopeptides (intermittent) | 22 | 7 | 13 | 2 |
| **Vascular access**, n (%) |  |  |  |  |
| PICC-line or Port | 190 (73) | 100 | 60 | 30 |
| PVC | 70 (27) | 21 | 47 | 2 |

**Notes:** An OPAT episode may have consisted of the administration of multiple antimicrobials simultaneously. Patients were allowed to be included several times in this study, by this, the data is presented as number of episodes.

**Abbreviations**: Outpatient parenteral antimicrobial therapy, OPAT; Peripherally inserted central catheter, PICC; Peripheral venous catheter, PVC; body mass index, BMI; Interquartile range, IQR

References

1. Stemkens R, Schouten JA, van Kessel SAM, Akkermans RP, Telgt DSC, Fleuren H, et al. How to use quality indicators for antimicrobial stewardship in your hospital: a practical example on outpatient parenteral antimicrobial therapy. Clin Microbiol Infect. 2023;29(2):182-7. <https://doi.org/10.1016/j.cmi.2022.07.007>.
